# Evaluation on Infectivity of *Babesia microti* to Domestic Animals and Ticks Outside the Ixodes Genus

**DOI:** 10.3389/fmicb.2017.01915

**Published:** 2017-10-06

**Authors:** Jiajun Wu, Jie Cao, Yongzhi Zhou, Houshuang Zhang, Haiyan Gong, Jinlin Zhou

**Affiliations:** ^1^Key Laboratory of Animal Parasitology of Ministry of Agriculture, Shanghai Veterinary Research Institute, Chinese Academy of Agricultural Sciences, Shanghai, China; ^2^Jiangsu Co-innovation Center for Prevention and Control of Important Animal Infectious Diseases and Zoonoses, Yangzhou, China

**Keywords:** *Babesia microti*, experimental infection, domestic animals, tick, *Haemaphysalis longicornis*

## Abstract

Babesiosis caused by *Babesia microti* parasite is an emerging tick borne zoonotic disease that was confirmed recently in China. To understand the epidemiology characteristics of this emerging disease, infectivity of *B. microti* to domestic animals and ticks outside the genus Ixodes was evaluated in this study. Different domestic animals, chick, pig, goat, dog and the reference host rat were experimentally inoculated with *B. microti*-infected erythrocytes and the parasite infection was monitored daily by blood smear observation, real-time PCR detection, nested-PCR and special antibody responses during 55 days period. The results showed that rats infected with *B. microti* showed a typical sustained infection with strongly antibody responses; however, both goats and dogs infected with *B. microti* only showed transient antibody responses and the parasite was not found by blood smear observation or PCR; neither the parasite nor the special antibodies were detected in experimental chicks and pigs. On the other hand, the present study also experimentally investigated the infectivity of *B. microti* to *Rhipicephalus haemaphysaloides, Haemaphysalis longicornis*, and *Hyalomma asiaticum* three species of ticks outside the genus Ixodes and the transmission experiment of *B. microti* between *H. longicornis* ticks and mice. Results showed that *B. microti* can be detected in the nymph and adult of these species after molting from engorged tick fed on infected mice, but the parasite was not detected in larvae hatching from eggs of engorged female tick fed on the infected mice. Transmission of *B. microti* to mice by infected *H. longicornis* nymphs was confirmed. These results indicated that these domestic animals do not have reservoir competence for *B. microti*, however, three species of ticks out of the genus Ixodes, common in China were successfully infected by *B. microti*, with *H. longicornis* showing the potential of transmitting the parasite to the vertebrate host.

## Introduction

*Babesia microti* are tick-borne protozoan parasites from the phylum Apicomplexa, which invade and replicate in RBCs of animals, including humans, causing zoonotic babesiosis (Gray et al., 2010). Thousands of cases of human babesiosis are reported in the world (Gray et al., 2010; Centers for Disease Control and Prevention [CDC], 2012; Fang et al., 2015), most caused by tick bites and also as a result of transferring infected blood; severe disease can result in susceptible patients, such as those who are splenectomized (Vannier and Krause, 2009). Most cases of babesiosis occur in the USA and parts of Europe, with fewer in Africa, Australia, Asia and other regions (Gray et al., 2010). In China, *B. microti* organisms have been found in local ticks (Sun et al., 2008), wild mice (Zhao et al., 2013) and human (Zhou X. et al., 2013) and was regarded as emerging public health threat (Fang et al., 2015; Vannier and Krause, 2015).

*B. microti* can infect a range of animal species, some of hosts were confirmed by experimental infection, and most of the others were deduced from the DNA detection in host samples or the blood feeding tick samples. Experimentally, some rodent species have shown susceptibility to infection with *B. microti* (Konopka and Sinski, 1996). Meanwhile intravenously inoculation *B. microti* showed the persistent infection with low parasitemia in rhesus macaques (van Duivenvoorde et al., 2010). In a recent study of reservoir competence in 14 wildlife host species (10 mammals and four birds), all species were regarded to be the reservoir hosts for *B. microti* by detecting blood feeding ticks on the hosts, which included rodents, raccoon and birds (Hersh et al., 2012). Infection with *B. microti* has also been observed in other wildlife mammal species, such as short tailed shrews (*Blarina brevicauda*) (Telford et al., 1990), eastern cottontail rabbits (*Sylvilagus floridanus*) (Spielman et al., 1981), and foxes (Birkenheuer et al., 2010). *B. microti* infection in birds has not been extensively studied*;* however, evidence of *B. microti* infection in birds had been discovered recently in Europe, which showed *B. microti* infection in engorged larval ticks that had been feeding on birds of several species (Franke et al., 2010a,b; Hildebrandt et al., 2010).

*B. microti* has long been known to infect both humans and rodents. Reservoir competence of wild animals for *B. microti* has been investigated extensively, however, the evidence whether *B. microti* can infect domestic animals such as pig, goat, dog and chick unclear yet. The Reservoir competence of domestic animals for *B. microti* will help advance understanding of the spread of human Babesiosis.

Tick species of the genus Ixodes are considered the primary vector of *B. microti* (Gray et al., 2010). *Ixodes scapularis* and *I. ricinus* are the main vectors of *B. microti* in the United States and Europe respectively (Oliveira and Kreier, 1979; Gray et al., 2002). In Asia, *B. microti* have been detected in questing tick *I. ovatus* and *I. persulcatus* (Sun et al., 2008; Zamoto-Niikura et al., 2012; Tuvshintulga et al., 2015), and a recent study confirmed *I. persulcatus* ticks as a vector for *B. microti* by experimental transmission (Zamoto-Niikura et al., 2016). In China, *I. persulcatus* is considered the primary vector of *B. microti*, which is distributed in the northeastern regions (Sun et al., 2008), however, up to date, *B. microti* infection case in China were all found in the south of China, where *I. persulcatus* ticks have not been found (Yao et al., 2012; Zhou X. et al., 2013). Therefore, competent vectors of *B. microti* in south of China are not yet well understood. Considering the dominant tick species in southern China, recently a preliminary experimental transmission of *B. microti* by *Rhipicephalus haemaphysaloides* ticks was carried out and found the transmission potential of this tick species (Li et al., 2016). *B. microti* has also been detected in ticks other than the species of genus Ixodes in the field (Wojcik-Fatla et al., 2012) and under experimental condition (Kusakisako et al., 2015). Therefore, more studies are needed on the transmission potentials of tick species from other genera.

## Materials and Methods

### Protozoa, Ticks and Experimental Animals

The strain of *B. microti* was supplied by American Type Culture Collection (ATCC) under the number ATCCR PRA-99^TM^ and was maintained in our laboratory by serial passage of parasitized erythrocytes in mice (Zhao et al., 2016). The colony of *R. haemaphysaloides* ticks was derived from water buffalo in Hubei Province in southern China (Zhou et al., 2006) and the colony of *Hyalomma asiaticum* ticks was derived from sheep in the Xinjiang Autonomous Region in northern China (Li et al., 2014). The colony of parthenogenetic *Haemaphysalis longicornis* ticks was derived from deer in the Shanghai Wildlife Park in China (Zhou J. et al., 2013). A single engorged female was used to establish each tick colony. Tick rearing was performed in the laboratory in a dark incubator at 25°C with 92% relative humidity and feeding on a New Zealand white rabbit. After three generations had been maintained under laboratory conditions, the colonies of ticks were established in Shanghai Veterinary Research Institute, Chinese Academy of Agricultural Sciences, Shanghai, China. According to general principles, sexual maturity animals were chosen for this study. Six-week-old KM mice (Shanghai Silaike Experimental Animal Co. Ltd.). Two-month-old SD rats (Shanghai Silaike Experimental Animal Co. Ltd.), Three-week-old Yellow hair chicks (Shanghai Songlian Experimental Animal Co. Ltd.), 2-month-old Changshang pigs (Shanghai Nongxi Pig Farm), 3-month-old Berger’s dogs (Shanghai Songlian Experimental Animal Co. Ltd.), and 1-month-old goats (Shanghai Songlian Experimental Animal Co. Ltd.) were used in this study. The experimental animals were treated following the approved guidelines from the Animal Care and Use Committee of the Shanghai Veterinary Research Institute (Approval number SHVRI-MO-0023), all surgery was performed under sodium pentobarbital anesthesia, and all efforts were made to minimize suffering.

### Inoculation of *B. microti*-Infected Erythrocytes in Animals

Blood containing *B. microti*-infected erythrocytes were collected by cardiac puncture from mice infected with *B. microti.* After measuring the total erythrocyte count and infection rate, parasitized erythrocytes were adjusted to 1 × 10^8^/mL by phosphate buffered saline (0.01M PBS, pH7.4). The *B. microti-*infected erythrocytes were then injected either intraperitoneally for rat or intravenously for chick, pig, dog, and goat with 1 × 10^7^ parasitized erythrocytes, three individuals were infected for each kinds of domestic animals. Blood samples were then collected from the animals once every 5 days until 55 days after inoculation.

### Microscopic Observation of *B. microti* in Blood Smears

Thin blood smears with Giemsa staining were used to examine *Babesia* parasites in infected red blood cells (RBCs) under a light microscope, a method that is currently used in most diagnostic laboratories (Mosqueda et al., 2012). The peripheral bloods from infected animals were taken, and then made thin blood smears with Giemsa staining for microscopic observation.

### Detection of *B. microti* in Animal Blood by Quantitative PCR and Nested-PCR

DNA was extracted from 300-μl of EDTA preserved whole blood using the QIAamp DNA minikit blood and body fluids protocol (Qiagen) according to the manufacturer’s instructions and eluted in an equal volume of elution buffer. Extracted DNA (5 μl) was used as a template for quantitative PCR following the methodology (Rollend et al., 2013), Forward and reverse primers and probe sequences are: Bm18Sf-AACAGGCATTCGCCTTGAAT, Bm18Sr-CCAACT GCTCCTATTAACCATTACTCT, and Bm18Sp-6FAM-CTA CAGCATGGAATAATGA-MGBNFQ, respectively. The quantitative PCR was performed using ABI 7500 instruments. Nested-PCR was used to detect parasites in blood samples by a previously published protocol (Persing et al., 1992).

### Detection of Antibody to *B. microti* in Host Animals after Infection

Whole blood samples were collected regularly from infected animals and the sera were collected and kept at -30°C. Enzyme-linked immunosorbent assay (ELISA) was carried out to detect the antibody responses against *B. microti* infection, which used the recombinant BmSA1 as the coated antigen (Luo et al., 2011). Briefly, 96-well microplates were coated with the antigen, glutathione *S*-transferase (GST)- BmSA1 and GST (negative control) at a concentration of 250 ng/well. Animal sera diluted at 1:100 with PBS containing 0.05% Tween 20 were used as the source of primary antibody and HRPO-conjugated anti-chicken IgG (ID, A30-107P) or anti-pig IgG (ID, A100-105P), or anti-goat IgG (ID, A50-100P), or anti-dog IgG (ID, A40-123P), or anti-rat IgG (ID, A110-105P) was used as the second antibody respectively (Bethyl Laboratories, United States). After addition of substrate [2,20-azino-bis (3-ethylbenzthiazoline-6-sulfonic acid)], the optical density (OD) value was recorded at 415 nm by SpectraMax M5 (Molecular Devices, United States).

### Blood Examination of the Infected Animals

EDTA-preserved blood was collected from infected animals as described above, blood examination was carried out using the Mindray BC-2800 Auto Hematology Analyzer (Mindray, China). Number of white blood cells (WBC), number of RBCs, hematocrit (HCT), and mean corpuscular volume (MCV) were checked.

### Ticks Infection by Blood Feeding on Mice Infected with *B. microti*

Approximately 600 larvae were applied equally to feed on 3 mice with *B. microti* parasitemia of 20%. Each mouse was collared with a plastic cap with central drilling to prevent grooming and then ticks were allowed to feed to repletion on infected mice. Parts of engorged larvae ticks were pooled (10 larvae ticks) for *B. microti* qPCR detection at once; the other engorged larvae were allowed to molt in the dark at 25°C, 92% relative humidity for 2–4 weeks. Newly molted tick nymphs were collected (individual) and used for qPCR detection. In the same way, about 100 nymphs were applied equally to feed on 3 mice with *B. microti* parasitemia of 20%. Some of the engorged nymphs and newly molted adult ticks from the engorged nymphal were collected (individual) and used for *B. microti* qPCR detection respectively. Also, about 60 adult female ticks (add 60 adult male ticks for *R. haemaphysaloides* and *H. asiaticum*) were applied equally to feed on 10–30 mice with *B. microti* parasitemia of 20% depending on different species of ticks. Some of engorged female ticks (individual) and the pool (10 larvae ticks) of newly molted larval ticks from the single engorged female were collected and used for *B. microti* qPCR detection.

### Transmission of *B. microti* to Mice by Infected *H. longicornis* Nymphs

Ten KM mice were infested with 60 nymphs each, which were previously infected with *B. microti* as larvae. Approximately 50 μl tail bloods of mice were collected for parasite detection every 3 days starting from day 7 to day 30 after tick infestation. When no *B. microti* infection was detected after blood samples were microscopically examined, each mouse was sacrificed, bled and a fresh mice was inoculated with the blood. The monitoring process was conducted again as mentioned above for another 30 days until positive mice were found.

For each blood sample, a thin blood smear was stained with Giemsa-stain in order to examine parasites by microscope. Forty-five microliters of blood were used for DNA extraction, and qPCR was performed to detect *B. microti* infection. Blood collection was stopped when the mice were proved to be infected upon microscopic examination.

### Data Analysis

Differences in the positive rate of ticks were tested by Chi-square, which was performed using SPSS 20.0 software. A probability *P*-value < 0.05 was considered statistically significant.

## Results

### Observation of Parasitemia by Blood Smears

Following infection with *B. microti*, parasitemia increased rapidly in rats, parasites were observed in RBCs 1 day after infection, with parasitemia peaking at 29.1% at day 6, then declining rapidly to <1% at day 11 post-infection (**Supplementary Figure S1**). Although it was difficult to detect parasites in RBCs of infected rats after day 11 post-infection, a few parasites could still be observed over the following few days. By contrast, no parasites were observed in the RBCs of dogs, goat, pigs and chicks at any time in the experiment periods (data not shown).

### Detection of the Parasite DNA by Quantitative PCR and Nested-PCR

As shown in **Table 1**, the parasite DNA could not be detected by quantitative PCR and nested-PCR in the blood samples of all host animals from 5 to 55 days after infection except the rat. The copy numbers of target gene of *B. microti* were never less than 10^3^ at any point during the experiment in the rat in real-time PCR (**Supplementary Figure S2**). However, *B. microti* did not to be detected in the blood samples of dogs, goats, pigs and chicks after 5 days following initial infection by both qPCR and nested-PCR (data not shown).

**Table 1:** Blood detection of animals infected with *B. microti* in 2 month periods.

Methods	Rat	Dog	Goat	Pig	Chick
Blood smear	+/-	–	–	–	–
Real-time PCR	+	–	–	–	–
Nested-PCR	+	–	–	–	–

*“+” indicates the positive result, “-” indicates the negative result.*

### Antibody Detection of Host Animals after Infections

**FIGURE 1:**
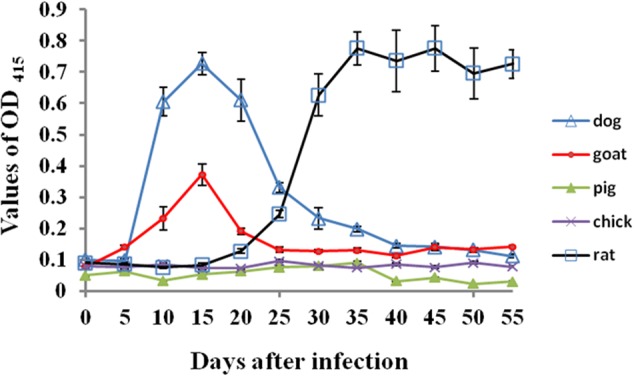
The specific antibody responses against *B. microti i*n different host animals post-infection. The error bars represent three biological replicates.

As shown in **Figure 1**, compared with rat serum before the infection, the specific antibodies against *B. microti* were detectable in rats as early as 10 days post-infection and a significant antibodies levels were found on 20 days post-infection, peaking at 30 days post-infection (OD > 0.8) and then kept high levels until the end of experiments. In dogs and goats, unexpectedly, antibody response was quick. Significant antibody levels were found on 10 days post-infection, peaking at 15 days post-infection and then antibody responses decreased quickly and disappeared on 20 and 30 days post-infection for goats and dogs respectively. By contrast, all serum samples from the experimental pigs and chicks showed no any specific antibody responses against *B. microti.*

### Blood Examination of the Infected Animals

**FIGURE 2:**
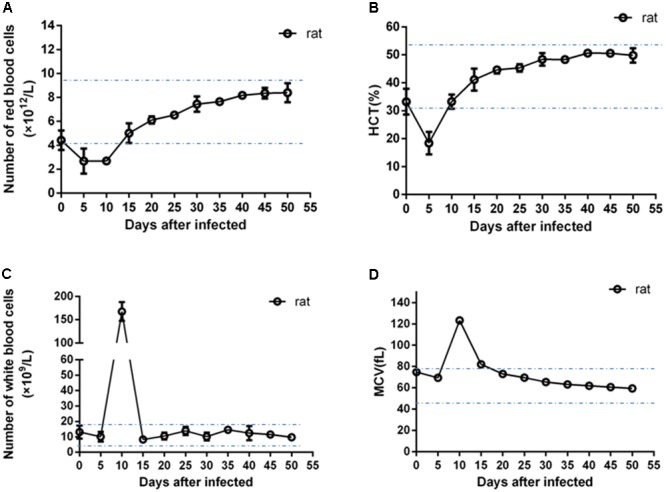
Blood examination of the infected rats. The error bars represent three biological replicates. The dotted lines showed the minimum and maximum values of normal ranges. **(A)** Values of number of red blood cells; **(B)** values of hematocrit (HCT); **(C)** values of number of white blood cells; **(D)** values of mean corpuscular volume (MCV).

As shown in **Figure 2**, during early infection in rats, the values of RBC and HCT were below the value of the normal range at day 5 post-infection. The numbers of RBCs of infected rats increased at 10 days post-infection and returned to the normal range at 15 days; the values of HCT returned to the normal range at 10 days post-infection. The numbers of leukocytes increased rapidly at day 5 post-infection, peaking at day 10 post-infection which was eight times the maximum normal value (20.9 × 10^9^ /L), returning to within the normal range at day 15 post-infection. The mean value of MCV of infected rats was also higher than the normal range at day 10 post-infection and returned normal at day 15 post-infection. By contrast, no significant changes from normal values were recorded in the levels of RBC, HCT, WBC and MCV in experimental infected dogs, goats, pigs and chicks (**Supplementary Figures S3–S6**).

### Ticks Infection by Blood Feeding on Mice Infected with *B. microti*

Tick larvae were fed on the mice infected with *B. microti* for 3–5 days, 600–650 larvae of *H. longicornis, R. haemaphysaloides* and *H. asiaticum* were engorged and collected respectively. As shown in **Table 2**, engorged larvae pools of three species of ticks were all 100% positive (20/20) by PCR, which indicated that the larvae ticks had ingested *B. microti* successfully. However, positive rates of molted ticks (nymphs) were 93.3, 86.7, and 83.3% for *H. longicornis, R. haemaphysaloides* and *H. asiaticum* respectively, which showed no significant difference between different species of ticks.

**Table 2:** Infection of larvae ticks by exposure to mice infected with *B. microti.*

Tick species	No. of engorged tick pools analyzed	Positive rate of engorged tick pool %s	No. of molted tick pools analyzed	Positive rate of molted tick pools %
*H. longicornis*	20	100	30	93.3
*R. haemaphysaloides*	20	100	30	86.7
*H. asiaticum*	20	100	30	83.3

About 150 engorged nymphs were collected from infected mice after feeding of *H. longicornis, R. haemaphysaloides*, and *H. asiaticum*. All 20 of the engorged nymphs tested by PCR were positive, however, positive rates of molted ticks (adult) were 70, 55, and 50% for *H. longicornis, R. haemaphysaloides*, and *H. asiaticum* respectively (**Table 3**), which showed significant difference between *H. longicornis* and the other two species of ticks. This result may suggest the different ability for transmission in this developmental stage.

**Table 3:** Infection of nymphal ticks by exposure to mice infected with *B, microti.*

Tick species	No. of engorged ticks analyzed	Positive rate of engorged ticks %	No. of molted ticks analyzed	Positive rate of molted licks %
*H. longicornis*	20	100	20	70^∗^
*R. haemaphysaloides*	20	100	20	55
*H. asiaticum*	20	100	20	50

*^∗^*P*-value < 0.05.*

About 50 engorged females of each tick species were collected from infected mice after feeding. All 20 of the engorged female ticks tested by PCR were positive, but no positive larvae tick pools from single female ticks were found in all tested samples in *H. longicornis, R. haemaphysaloides*, and *H. asiaticum* (**Table 4**).

**Table 4:** Infection of adult ticks by exposure to mice infected with *B. microti.*

Tick species	No. of engorged ticks analyzed	Positive rate of engorged ticks %	No. of larvae tick pools from single female analyzed	Positive rate of Larvae tick pools from single female
*H. longicornis*	20	100	20	0
*R. haemaphysaloides*	20	100	20	0
*H. asiaticum*	20	100	20	0

### Transmission of *B. microti* to Mice by Infected *H. longicornis* Nymphs

As shown in **Table 5**, most of infected nymphs had successfully engorged on mice between 3 and 5 days. However, *B. microti* was not detected in mice in the first 30 days after tick infestation and the second 30 days after collected blood was blindly passed by inoculation. Parasites were detected by qPCR in 2 out of 10 mice in the third 30 days after blood passage. The parasite was not found in the blood smears of mice until the fourth 30 days after blind passage of blood (**Supplementary Figure S7**). It took about 4 month to firstly detect *B. microti* infection after tick infestation test.

**Table 5:** Transmission of *B. microti* to mice by infected *H. longicornis* nymphs.

Mice	No. of engorged /infected nymphs	Days after infestation for qPCR positive	Days after infestation for blood smear positive
1	45/60		
2	40/60	90	103
3	35/60	113	
4	48/60		
5	32/60		
6	44/60	90	113
7	52/60		
8	43/60		
9	50/60	120	
10	51/60	117	

## Discussion

As the infection reference for *B. microti* inoculation, the rats showed a typical infection course throughout the experimental periods similar to mice or hamster infection (Konopka and Sinski, 1996; Ike et al., 2005). *B. microti* can persist in rats over long time and with high antibody response levels. Moreover, during early stages of *B. microti* infection, the numbers of RBC and HCT of infected rats were significantly lower than the normal range, and the number of MCV was higher than the normal range, which suggests that rats would display symptoms of anemia. In addition, a significant finding was that the number of WBC increased to several times the normal value in rats at 10 days post-infection.

However, this study showed that the goat, pig, dog, and chick were not infected with *B. microti* by systematic surveys on parasitological observation, DNA detection, and blood examination for physiology indexes. Although goats and dogs infected with *B. microti* showed transient antibody responses, the parasite was not found by blood smear observation or PCR; this transient antibody response might be caused by the dog and goat immune system against the inoculated parasites, but not in the chick and pig.

The intravenously inoculation is the common method for *B. microti* experimental infection (van Duivenvoorde et al., 2010), in this study, considering the convenience and reliability for rats, *B. microti*-infected erythrocytes were injected intraperitoneally. This inoculation route difference between rat and other domestic animals is not relative with successful infection.

Some reports have suggested that *B. microti*-like parasites exhibit infectivity in dogs (Zahler et al., 2000; Camacho et al., 2001), however, the evidence whether *B. microti* can infect dogs or not was not clear yet. In the present experiment, we examined the inoculation of mouse-derived *B. microti* in dogs. A transient antibody response was found in the early time after parasites inoculation. However, the following evidences (parasitological observation, DNA detection, blood examination for physiology indexes) strongly suggested that dogs were not infected with this parasite in present study.

Evidence of *B. microti* infection in wild birds had recently been discovered in engorged larval ticks (or newly molted nymphs) that had been feeding on birds of several species (Franke et al., 2010b; Hildebrandt et al., 2010; Capligina et al., 2014). Unexpectedly, chicks as domestic birds were not proved to be infected in these studies. The *B. microti* DNA was only detected in 5 days post inoculation samples, while possible inoculation remnant was not found in any other time point in whole experimental course. Meantime, there were no significant antibody responses against *B. microti* infection, both microscopic observation and blood examination showed negative infection in chicks.

Ticks genera other than the genus Ixodes, such as *Rhipicephalus* genus, *Haemaphysalis* genus are very common and widespread in southern China. Dozens of human infections with *B. microti* have been reported recently, especially in southern China (Vannier and Krause, 2015). The vectors of this parasite in these areas are not well understood, and the transmission capability of ticks outside the genus Ixodes for *B. microti* need to be elucidated. In this study, the three species of tick from three genera were tested and all showed the transmission potential competence. Our results confirmed recent report on the potential vector of *R. haemaphysaloides* (Li et al., 2016). Moreover, according to positive rate of molted ticks (nymphal and adult), *H. longicornis* tick is more potentially competent for *B. microti* transmission, which is confirmed by the transmission tests of *B. microti* to mice by infected *H. longicornis* nymphs. This result showed some different from the report on vector potential of *H. longicornis* ticks for *B. microti* (Kusakisako et al., 2015), which indicate that *B. microti* can infect to this kind of ticks, but the transmission was not confirmed. The unsuccessful transmission experiment might be involved in many factors of study; one of the key factors may come from the short period (0–40 days) to detect the parasite. In this study, *B. microti* positive detection was occurred firstly on the 90th days after tick infestation by qPCR and the 103th days after tick infestation by blood smear examination. Because blood collection was stopped when the mice were proved to be infected upon microscopic examination, more positive infection might be found later than 120 days after infection. To our knowledge, this is the first experiment report to show vector competence of the *H. longicornis* tick for *B. microti* transmission.

## Conclusion

We explored the infectivity of *B. microti* parasites in domestic animals and ticks other than the genus Ixodes, and found that dogs, goat, pigs and chicks were not the reservoir hosts, however, ticks investigated in this study *R. haemaphysaloides,H. longicornis* and *H. asiaticum* all showed potential competence for *B. microti* transmission.

## Author Contributions

JZ directed the project, participated in the design of the study, and helped write manuscript. JW and JC performed the laboratory tests, analyzed the data, and wrote the manuscript. YZ participated in the animal experiments. HG and HZ participated in data interpretation.

## Conflict of Interest Statement

The authors declare that the research was conducted in the absence of any commercial or financial relationships that could be construed as a potential conflict of interest.

## Supplementary Material

FIGURE S1 Parasitemia in the infected rats. The error bars represent three biological replicates.

FIGURE S2 DNA detection in the infected rats. The error bars represent three biological replicates.

FIGURE S3 Blood examination of the infected chicken. The error bars represent three biological replicates.

FIGURE S4 Blood examination of the infected pig. The error bars represent three biological replicates.

FIGURE S5 Blood examination of the infected dog. The error bars represent three biological replicates.

FIGURE S6 Blood examination of the infected goat. The error bars represent three biological replicates.

FIGURE S7 The parasites in the blood smear of mouse experimentally transmitted by *H. longicornis* nymphs. The arrows indicated the parasites in RBC of mouse.
