# Supplementary figures and images for: Evaluation on Infectivity of Babesia microti to Domestic Animals and Ticks Outside the Ixodes Genus
Source: Front Microbiol. 2017 Oct 6;8:1915. doi: 10.3389/fmicb.2017.01915 (PMC5635051; doi:10.3389/fmicb.2017.01915)

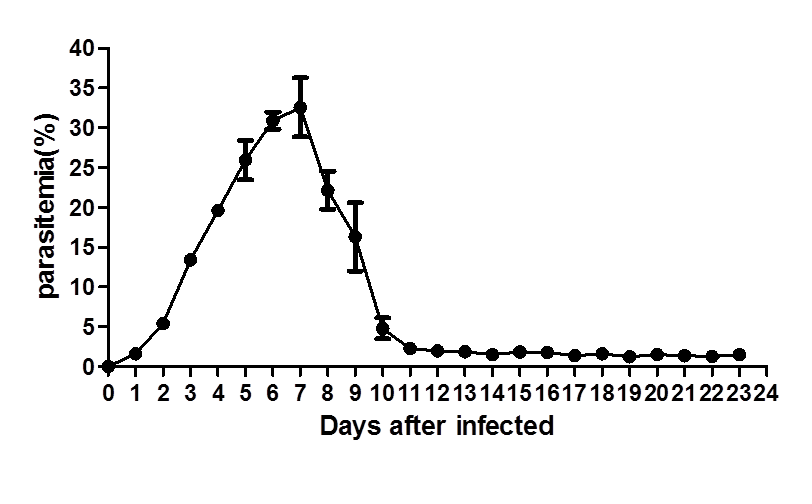

Supplement: FIGURE S1 — Parasitemia in the infected rats. The error bars represent three biological replicates. [file Image_1.TIF]

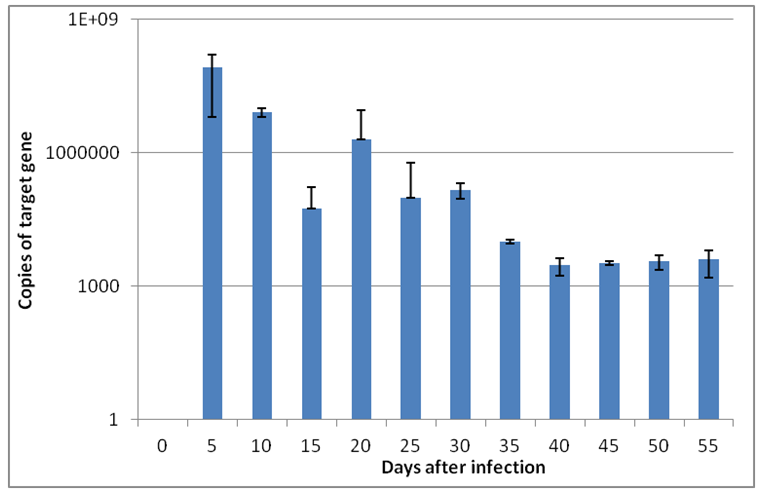

Supplement: FIGURE S2 — DNA detection in the infected rats. The error bars represent three biological replicates. [file Image_2.TIF]

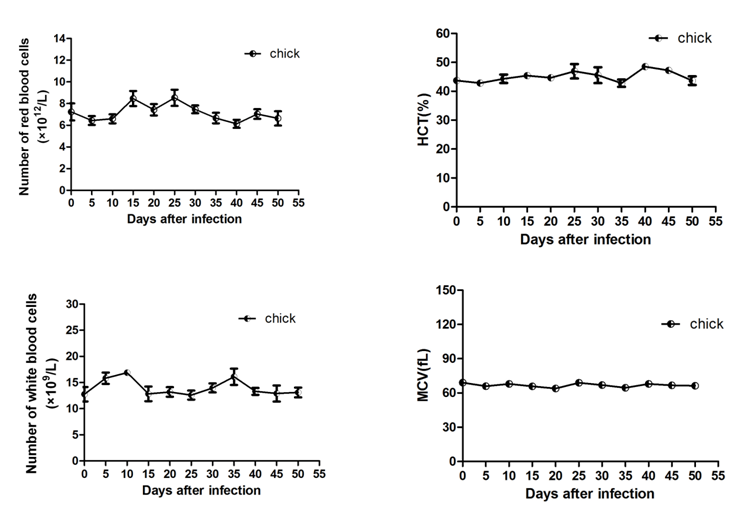

Supplement: FIGURE S3 — Blood examination of the infected chicken. The error bars represent three biological replicates. [file Image_3.TIF]

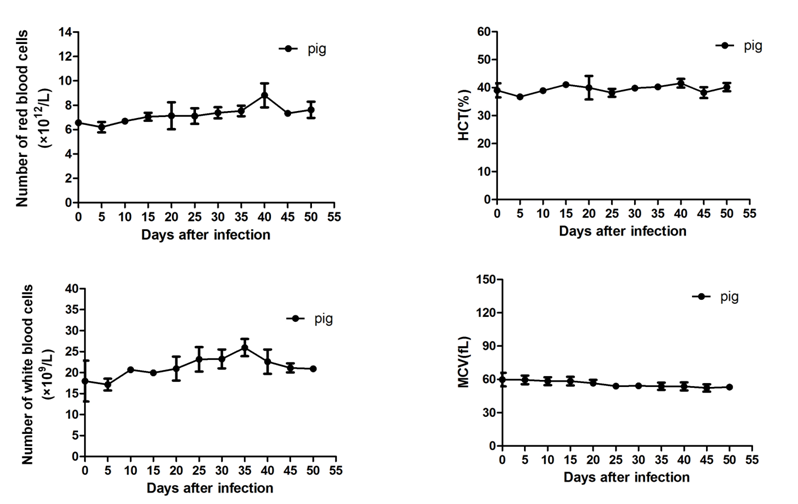

Supplement: FIGURE S4 — Blood examination of the infected pig. The error bars represent three biological replicates. [file Image_4.TIF]

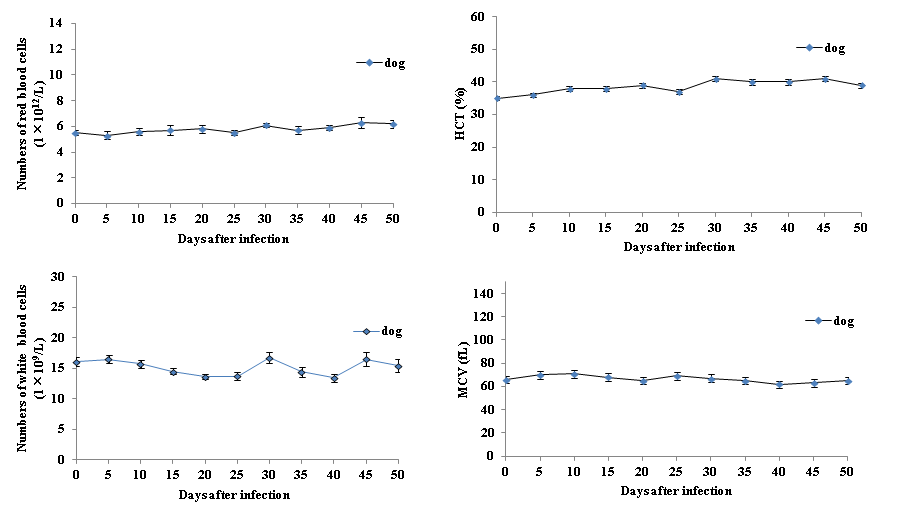

Supplement: FIGURE S5 — Blood examination of the infected dog. The error bars represent three biological replicates. [file Image_5.TIF]

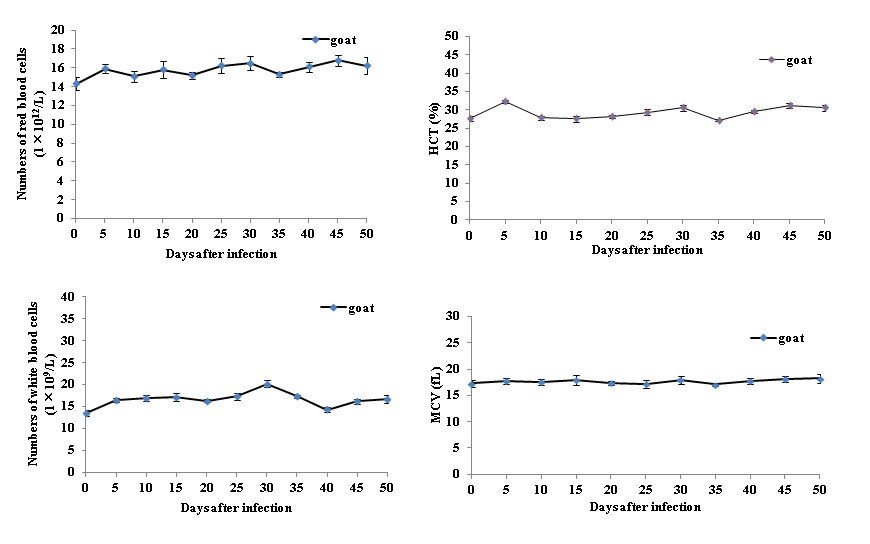

Supplement: FIGURE S6 — Blood examination of the infected goat. The error bars represent three biological replicates. [file Image_6.TIF]

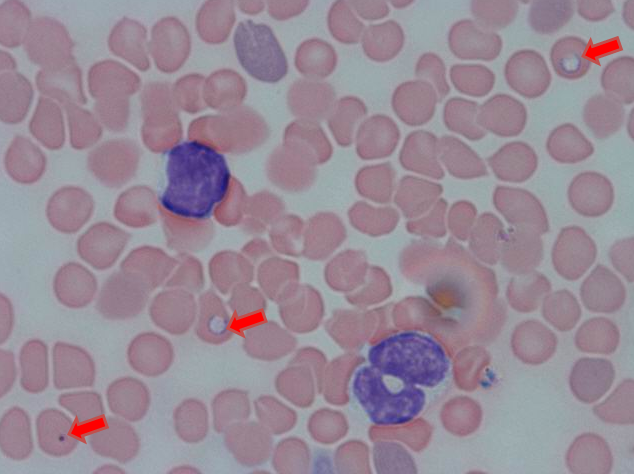

Supplement: FIGURE S7 — The parasites in the blood smear of mouse experimentally transmitted by H. longicornis nymphs. The arrows indicated the parasites in RBC of mouse. [file Image_7.TIF]
